# Single Nucleotide Polymorphisms within *LIPA* (Lysosomal Acid Lipase A) Gene Are Associated with Susceptibility to Premature Coronary Artery Disease. A Replication in the Genetic of Atherosclerotic Disease (GEA) Mexican Study

**DOI:** 10.1371/journal.pone.0074703

**Published:** 2013-09-17

**Authors:** Gilberto Vargas-Alarcón, Carlos Posadas-Romero, Teresa Villarreal-Molina, Edith Alvarez-León, Javier Angeles, Maite Vallejo, Rosalinda Posadas-Sánchez, Guillermo Cardoso, Aida Medina-Urrutia, Eric Kimura-Hayama

**Affiliations:** 1 Department of Molecular Biology, Instituto Nacional de Cardiología Ignacio, Chávez, Mexico City, Mexico; 2 Department of Endocrinology, Instituto Nacional de Cardiología Ignacio, Chávez, Mexico City, Mexico; 3 Cardiovascular Genomics Laboratory, Instituto Nacional de Medicina Genómica (INMEGEN), Mexico City, Mexico; 4 Sociomedical Department, Instituto Nacional de Cardiología Ignacio, Chávez, Mexico City, Mexico; 5 Department of Tomography, Instituto Nacional de Cardiología Ignacio, Chávez, Mexico City, Mexico; Medical University Hamburg, University Heart Center, Germany

## Abstract

**Aim:**

The rs1412444 and rs2246833 polymorphisms within the *LIPA* gene were recently found to be significantly associated with coronary artery disease (CAD) in genome-wide association studies in Caucasian and Asian populations. The aim of the present study was to replicate this association in an independent population with a different genetic background.

**Methods:**

The rs1412444 and rs2246833 polymorphisms of the *LIPA* gene were genotyped by 5′ exonuclease TaqMan genotyping assays in a sample of 899 Mexican patients with premature CAD, 270 individuals with subclinical atherosclerosis, and 677 healthy unrelated controls. Haplotypes were constructed after linkage disequilibrium analysis.

**Results:**

Under recessive and additive models, the rs1412444 *T* and rs2246833 *T* alleles were associated with an increased risk of premature CAD when compared to controls adjusting for age, gender, BMI, and total cholesterol (OR = 1.53, P_Rec_ = 0.0013 and OR = 1.34, P_Add_ = 5 × 10^-4^ for rs1412444 and OR = 1.45, P_Rec_ = 0.0039 and OR = 1.28, P_Add_ = 0.0023 for rs2246833). The effect of the two polymorphisms on various metabolic cardiovascular risk factors was analyzed in premature CAD and controls (CAC score = 0). The *T* alleles in both polymorphisms after adjusting for age, gender, BMI, and medication were associated with hypo-α-lipoproteinemia, hypercholesterolemia, hypertriglyceridemia, metabolic syndrome, and type 2 diabetes mellitus using recessive and additive models. The polymorphisms were in strong linkage disequilibrium and, based on SNP functional prediction software, only the rs1412444 polymorphism seemed to be functional.

**Conclusions:**

These results indicate that the rs1412444 and rs2246833 of the *LIPA* gene are shared susceptibility polymorphisms for CAD among different ethnicities.

## Introduction

Coronary artery disease (CAD) is a complex multifactorial and polygenic disorder resulting from an excessive inflammatory response to various forms of injurious stimuli to the arterial wall [1-3]. Although the precise mechanisms responsible for the onset of the disease are still unknown, multiple genetic factors may cooperate with environmental factors to confer susceptibility to CAD. Genome wide association (GWA) studies have identified several genetic loci associated with the risk of CAD in different ethnic groups [4-9]. Results of association studies may vary between populations due to interpopulation genetic differences, including differences in allele frequencies and linkage disequilibrium (LD) structures [10]. Therefore, it is important to examine multiple ethnic populations through GWA studies for the identification of ethnicity-specific loci as well as common susceptibility loci. Recent GWA studies followed by two states of replication and a final meta-analysis in Caucasian individuals led to the identification of a novel CAD susceptibility locus on chromosome 10q23, 31, the *LIPA* (lysosomal acid lipase A) gene [11]. Monocyte gene expression analysis revealed an effect of *LIPA* single nucleotide polymorphisms (SNPs) on *LIPA* transcript levels. Two SNPs (rs1412444 and rs2246833) showed strong association with expression of the *LIPA* transcript levels. The *LIPA* gene encodes lysosomal acid lipase (LAL), which hydrolyzes cholesteryl esters and triglycerides in the lysosome of cells to generate free cholesterol and free fatty acids [12]. Any alteration of LAL could produce an accumulation of triglycerides and cholesterol esters in the cell, resulting in foam cells and, consequently, in atherosclerotic plaque formation [13]. Patients with LAL deficiency show lipid accumulation in cells and develop premature atherosclerosis [14]. The role of the *LIPA* locus in CAD was corroborated in another GWA study in Caucasian and Asian populations [9]. Considering these studies, the aim of a present work was to determine whether the association of two SNPs with CAD, reported in Caucasian and Asian populations, is replicated in Mexican patients with premature CAD. The analysis of independent populations with different ethnic background often helps to unravel the genetic implications in associated regions by narrowing down the linkage structure.

## Materials and Methods

All participants provided written informed consent, and the study complies with the Declaration of Helsinki and was approved by the Ethics Committee of the Instituto Nacional de Cardiología ‘‘Ignacio Chávez’’ (INCICH) and of the Instituto Nacional de Medicina Genómica. The primary aim of the GEA Study is to investigate genetic factors associated with premature CAD, subclinical atherosclerosis, and other coronary risk factors in the Mexican population.

### Subjects

All GEA participants are unrelated and of self-reported Mexican-Mestizo ancestry (3 generations). A Mexican Mestizo is defined as someone born in Mexico, who is a descendant of the original autochthonous inhabitants of the region and of individuals, mainly Spaniards, of Caucasian and/or African origin, who came to America during the sixteenth century. The study included 899 patients with premature CAD, 270 individuals with subclinical atherosclerosis (SA), and 677 healthy controls from the Genetic of Atherosclerotic Disease (GEA) Study. The selection of patients and controls of the GEA Study has been described previously [15]. Demographic, clinical, anthropometric, and biochemical parameters and cardiovascular risk factors were evaluated in patients and controls.

### Genetic analysis

Genomic DNA from whole blood containing EDTA was isolated by standard techniques. The rs1412444 and rs2246833 single nucleotide polymorphisms (SNPs) were genotyped using 5’ exonuclease TaqMan genotyping assays on an ABI Prism 7900HT Fast Real-Time PCR system, according to manufacturer’s instructions (Applied Biosystems, Foster City, CA, USA)

### Statistical analysis

All calculations were performed using SPSS version 18.0 (SPSS, Chicago, Il) statistical package. Means ± standard deviations (SD) and frequencies of baseline characteristics were calculated. Chi-square tests were used to compare frequencies and ANOVA and Student’s *t*-test were used to compare means. ANCOVA was used to determine associations between the polymorphisms and metabolic variables, adjusting for age, gender, BMI, triglycerides, alcohol consumption, and smoking, as appropriate. Logistic regression analysis was used to test for associations of polymorphisms with premature CAD under inheritance models. The most appropriate inheritance model was selected based on Akaike information criteria and was adjusted for age, gender, BMI, triglycerides, alcohol consumption, and smoking. To address multiple testing, Bonferroni’s correction was used considering five independent tests, and statistical significance was set at p < 0.01. The statistical power estimated with QUANTO software (http://hydra.usc.edu/GxE/) to detect association between premature CAD and controls was 0.96 for rs1412444 and 0.90 for rs2246833, whereas to detect association between premature CAD and SA was 0.90 for rs1412444 and 0.77 for rs2246833. The power to detect association between SA and controls was low (0.1 for rs1412444 and 0.08 for rs2246833). Genotype frequencies did not show deviation from Hardy-Weinberg equilibrium (HWE) (p > 0.05). Pairwise linkage disequilibrium (LD, D´) estimations between polymorphisms and haplotype reconstruction were performed with Haploview version 4:1 (Broad Institute of Massachusetts Institute of Technology and Harvard University, Cambridge, MA, USA).

### Functional prediction analysis

We predicted the potential effect of *LIPA* SNPs associated with premature CAD in our population using bioinformatics tools, including FastSNP [16], SNP Function Prediction (http://snpinfo.niehs.nih.gov/snpfunc.htm), Human-transcriptome Database for Alternative Splicing (http://www.h-invitational.jp/h-dbas/), SplicePort (http://www.spliceport.cs.umd.edu/SplicingAnalyser2.html), ESE finder

(http://rulai.cshl.edu/cgi-bin/tools/ESE3/esefinder.cgi), HSF

(http://www.umd.be/HSF), and SNPs3D (http://www.snps3d.org/).

## Results

### Characteristics of the study sample

General characteristics of the population are shown in Tables 1 and 2. Because 270 (28.8%) of the apparently healthy individuals recruited as controls showed a positive coronary calcium score, three independent groups were considered for the analysis: controls (CAC score = 0), subclinical atherosclerosis (CAC score > 0), and premature CAD.

**Table 1:** Demographic characteristic of the population.

			CONTROL	SA	Premature CAD	P*
			(n = 667)	(n = 270)	(n = 899)	
Age (years)			52.3 ± 9.1	58.7 ± 8.4	53.3 ± 7.4	< 0.0001
Gender (% Male)			38.1	72.6	82.6	< 0.0001
Body mass index (kg/m^2^)		28.4 ± 4.6		28.8 ± 4.5	28.7 ± 4.8	0.2920
Obesity (%)			47.3	47.4	36.6	< 0.0001
Awaist circunference (cm)		93.4 ± 11.7		97.4 ± 11.1	98.6 ± 11	< 0.0001
Central Obesity (%)		79.6		82.2	83.7	0.1030
Total Abdominal Fat (cm^2^)		452.5 ± 151.4		467.1 ± 159.5	442.5 ± 144.4	0.0520
Subcutaneous Abdominal Fat (cm^2^)		152.5 ± 66.9		189.4 ± 189.4	180.1 ± 180.1	< 0.0001
Visceral Abdominal Fat (cm^2^)		152.5 ± 66.9		189.4 ± 68.3	180.1 ± 73.3	< 0.0001
Visceral/Subcutaneous adipose tissue ratio		0.6 ± 0.3		0.8 ± 0.3	1.6 ± 0.8	< 0.0001
Current Smokers (%)		22.9		21.1	12.6	< 0.0001
Former Smokers (%)		31.6		45.2	64.4	< 0.0001
Hypertension (%)		54.5		50	67.1	< 0.0001
Hypertensive Medication (%)		15.4		28.5	66.8	< 0.0001
Diastolic Blood Pressure (mmHg)		72.8 ± 9.3		77.6 ± 10.6	73.1 ± 10.1	< 0.0001
Systolic Blood Pressure (mmHg)		118.3 ± 17.2		128.2 ± 20	119.7 ± 18.8	< 0.0001
Heart rate (bpm)		65.5 ± 8.9		66.2 ± 10.2	64.9 ± 11.5	0.2130

Data are expressed as means ± SD, log-transformed values were used for statistical analysis.

* P values were computed using ANOVA for continuous variables and Pearson’s Chi-square test for categorical values.

CAD: coronary artery disease; SA: subclinical atherosclerosis.

**Table 2:** Comparison of biochemical parameters in individuals with premature coronary artery disease, subclinical atherosclerosis, and controls.

		CONTROL	SA	Premature CAD	P*
		(n = 667)	(n = 270)	(n = 899)	
Total Cholesterol (mg/dl)		191.3 ± 35.9	198.3 ± 37.2	168.8 ± 47.8	< 0.0001
TC > 200 mg/dl (%)		35.3	47.4	21.7	< 0.0001
HDL-C (mg/dl)		48.3 ± 14.1	44.6 ± 11.7	40.2 ± 10.5	< 0.0001
Hipoa-lipoproteinemia (%)		50.1	48.5	63.6	< 0.0001
LDL-C (mg/dl)		116.4 ± 31.8	124.0 ± 31.03	97.6 ± 39.2	< 0.0001
Triglycerides (mg/dl)		165.5 ± 15.2	181.4 ± 107.4	192.9 ± 124.3	< 0.0001
Hypertriglyceridemia (%)		45.2	53.3	58.4	< 0.0001
ApoAI (mg/dl)		139.3 ± 41.1	138.9 ± 36.6	121.0 ± 26.4	< 0.0001
ApoB (mg/dl)		89.7 ± 27.5	98.2 ± 27.9	23.8 ± 16.3	< 0.0001
Statin and/or Fibrate treatment (%)		7.4	12.2	94.7	< 0.0001
Type 2 Diabetes mellitus (%)		10.0	22.6	35.4	< 0.0001
Glucose (mg/dl)		89.9 ± 9.3	93.0 ± 9.7	91.4 ± 11.4	0.0010
HOMA-IR		4.4 ± 2.7	4.7 ± 2.6	5.1 ± 3.3	< 0.0001
Alanine Transaminase (IU/l)		27.9±18.1	27.3±17.6	29.3±17.6	0.1350
Aspartate Transaminase (IU/l)		27.5 ± 11.3	28.6 ± 13.9	27.9 ± 10.9	0.4010
Alkaline Phosphatase (IU/l)		84.2 ± 23.7	82.1 ± 32.3	80.3 ± 25.6	0.0150
Gamma-glutamyl transpeptidase (IU/l)		34.7 ± 31.5	38.7 ± 34.5	45.1 ± 43.4	< 0.0001

Data are expressed as means ± SD, log-transformed values were used for statistical analysis.

* P values were computed using ANOVA for continuous variables and Pearson’s Chi-square test for categorical values.

CAD: coronary artery disease; SA: subclinical atherosclerosis.

### Association of polymorphisms with premature CAD

Observed and expected frequencies in the polymorphic sites were in HWE. The distribution of the rs1412444 and rs2246833 polymorphisms was similar in patients with subclinical atherosclerosis and healthy controls, but different in patients with premature CAD. The rs1412444 *T* allele was significantly associated with an increased risk of premature CAD as compared to controls under both recessive and additive models adjusting for age, gender, BMI, and total cholesterol (OR = 1.53, 95% CI: 1.18-1.99, P_rec_ = 0.0013 and OR = 1.34, 95% CI: 1.14-1.58, P_add_ = 5 × 10^-4^) and as compared to SA under dominant and additive models adjusted by the same variables (OR = 1.74, 95% CI: 1.23-2.48, P_dom_ = 0.0021 and OR = 1.31, 95% CI: 1.06-1.62, P_add_ = 0.0110). On the other hand, the rs2246833 *T* allele was significantly associated with increased risk of premature CAD as compared to controls under both recessive and additive models adjusted by age, gender, BMI, and total cholesterol (OR = 1.45, 95% CI: 1.12-1.87, P_rec_ = 0.0039 and OR = 1.28, 95% CI: 1.09-1.51, P_add_ = 0.0023) and as compared to SA under dominant and additive models adjusted by the same variables (OR = 1.60, 95% CI: 1.14-2.23, P_dom_ = 0.0071 and OR = 1.26, 95% CI: 1.03-1.54, P_add_ = 0.0230) (Table 3). The two *LIPA* polymorphisms were in strong linkage disequilibrium (D’ = 0.980, r^2^ = 0.937) forming four haplotypes (data not shown).

**Table 3:** Association of the (C > T) rs1412444 and (C > T) rs2246833 polymorphisms with premature coronary artery disease and subclinical atherosclerosis.

**rs1412444**	**GENOTYPE FREQUENCY (%**)						
	**C/C**	**C/T**	**T/T**	**RAF**	**MODEL**	**OR (95% CI**)	**P**
**CONTROL (n = 677**)	0.258	0.501	0.241	0.491			
**SA (n = 270**)	0.263	0.459	0.278	0.493			
**Premature CAD (n = 899**)	0.200	0.479	0.321	0.561	Recessive†	**1.53 (1.18-1.99)**	**0.0013**
					Additive†	**1.34 (1.14-1.58)**	**5X10^-4^**
					Dominant‡	**1.74 (1.23-2.48)**	**0.0021**
					Additive‡	**1.31 (1.06-1.62)**	**0.0110**
**rs2246833**	**GENOTYPE FREQUENCY (%**)						
	**C/C**	**C/T**	**T/T**	**RAF**	**MODEL**	**OR (95% CI**)	**P**
**CONTROL (n = 677**)	0.247	0.502	0.251	0.502			
**SA (n = 270**)	0.259	0.456	0.285	0.513			
**Premature CAD (n = 899**)	0.196	0.482	0.323	0.563	Recessive†	**1.45 (1.12-1.87)**	**0.0039**
					Additive†	**1.28 (1.09-1.51)**	**0.0023**
					Dominant‡	**1.60 (1.14-2.23)**	**0.0071**
					Additive‡	**1.26 (1.03-1.54)**	**0.0230**

Associations were tested using logistic regression adjusting for age, gender, BMI, and TC levels.

SA: subclinical atherosclerosis; CAD: coronary artery disease; RAF: risk allele frequency.

† Compared to controls.

‡ Compared to individuals with subclinical atherosclerosis.

No differences were observed when comparing SA individuals with controls.

### Association of the polymorphisms with metabolic cardiovascular risk factors and metabolic parameters

The effect of the two polymorphisms on various metabolic cardiovascular risk factors was analyzed in premature CAD and controls (CAC score = 0). The *T* alleles of both polymoprhisms after adjusting for age, gender, BMI, and medication were associated with hypo-α-lipoproteinemia, hypercholesterolemia, hypertriglyceridemia, metabolic syndrome, and type 2 diabetes mellitus using recessive and additive models (Tables 4 and 5). On the other hand, the effect of the polymorphisms on metabolic parameters was explored separately in controls (CAC score = 0), subclinical atherosclerosis (CAC score > 0), and premature CAD. No association with metabolic parameters was observed in any study group (data not shown).

**Table 4:** Association of the (C > T) rs1412444 polymorphism with coronary risk factors.

	**RAF Controls**	**RAF Premature CAD**	**MODEL**	**OR (95% CI**)	**P**
**Hypo-α-lipoproteinemia**	(n = 339)	(n = 572)	Recessive	2.50 (1.24-5.02)	0.0087
	0.492	0.548			
			Additive	1.90 (1.24-2.93)	0.0028
**Hypercholesterolemia**	(n = 245)	(n = 198)	Recessive	2.63 (1.03-6.75)	0.0370
	0.481	0.588			
			Additive	1.96 (1.12-3.41)	0.0180
**Hypertriglyceridemia**	(n = 306)	(n = 528)	Recessive	2.07 (1.10-3.88)	0.0200
	0.503	0.574			
			Additive	1.85 (1.26-2.72)	0.0015
**Metabolic Syndrome**	(n = 277)	(n = 428)	Recessive	2.57 (1.35-4.89)	0.0022
	0.503	0.554			
			Additive	1.94 (1.34-2.82)	0.0003
**Type 2 Diabetes Mellitus**	(n = 68)	(n = 325)	Recessive	2.64 (1.27-5.50)	0.0060
	0.455	0.578			
			Additive	1.83 (1.19-2.81)	0.0051

All association were tested using logistic regression adjusted for age, gender, BMI, and medication when appropriate. (n) Represents the number of cases with each trait.

RAF: risk allele frequency.

**Table 5:** Association of the (C > T) rs2246833 polymorphism with coronary risk factors.

	**RAF Controls**	**RAF Premature CAD**	**MODEL**	**OR (95% CI**)	**P**
**Hypo-α-lipoproteinemia**	(n = 339)	(n = 572)	Recessive	2.51 (1.25-5.04)	0.0079
	0.492	0.548			
			Additive	2.01 (1.31-3.10)	0.0011
**Hypercholesterolemia**	(n = 245)	(n = 198)			
	0.481	0.588	Additive	1.91 (1.10-3.34)	0.0200
**Hypertriglyceridemia**	(n = 306)	(n = 528)	Recessive	2.02 (1.08-3.79)	0.0230
	0.503	0.574			
			Additive	1.92 (1.30-2.83)	0.0008
**Metabolic Syndrome**	(n = 277)	(n = 428)			
	0.503	0.554	Additive	1.71 (1.19-2.45)	0.0033
**Type 2 Diabetes Mellitus**	(n = 68)	(n = 325)	Recessive	2.08 (1.03-4.18)	0.0330
	0.455	0.578			
			Additive	1.79 (1.17-2.74)	0.0062

All association were tested using logistic regression adjusted for age, gender, BMI, and medication when appropriate. (n) Represents the number of cases with each trait.

RAF: risk allele frequency.

## Discussion

In this study, we observed that two *LIPA* polymorphisms previously identified in a GWA study in Caucasian and Asian populations (rs1412444 and rs2246833) are also associated with premature CAD in the Mexican population [9,11]. It is important to point out that subclinical atherosclerosis was ruled out among controls by computed tomography, only including individuals with calcium score = 0. Individuals initially recruited as controls with calcium score > 0 were studied as an independent group with SA. It is noteworthy that, although in a previous study in Caucasians, these polymorphisms were also associated with *LIPA* expression and that this expression was related to subclinical disease assessed by vascular endothelial function [11]. In the present study no differences in allele frequencies were observed between controls and individuals with subclinical atherosclerosis assessed by computed tomography. However, this may be due to the low statistical power considering the sample size of the group of individuals with SA included in the study. On comparing this study with that reported by Wild et al. [11] there are some differences in study design. While Wild et al. included only patients with severe coronary atherosclerosis documented by angiography and myocardial infarction, our study included patients with unstable angina. In addition, we included only patients with premature CAD diagnosed before age 55 in men and age 65 in women. This suggests that *LIPA* polymorphisms are associated with CAD independently from the severity and age of onset of the disease.

The *LIPA* gene is a biological candidate for CAD and dyslipidemia because it is involved in cholesteryl ester and triglyceride hydrolysis in lysosomes to generate free cholesterol and free fatty acids, and, consequently, plays a role in atherosclerotic plaque formation. Moreover, individuals heterozygous for the E8SJM *LIPA* mutations were found to have altered lipid profiles with a polygenic hypercholesterolemia phenotype, leading to an increase in cardiovascular risk profile [17]. The association of the LIPA polymorphisms with metabolic and coronary risk factors had been analyzed previously with contradictory results. Wild et al. [11] reported that elevated LIPA expression was significantly associated with lower HDL-cholesterol levels and impaired endothelial function measured by flow-mediated vasodilatation, whereas associations with higher levels of LDL-cholesterol and triglycerides did not reach statistical significance. In contrast, no significant association between *LIPA* polymorphisms and any cardiovascular risk factor was observed. The IBC 50K CAD Consortium [18] reported no association between LIPA polymorphisms and altered lipid levels. We thus explored whether *LIPA* polymorphisms were associated with other metabolic cardiovascular risk factors, finding that these polymorphisms were significantly associated with hypo-α-lipoproteinemia, hypercholesterolemia, hypertriglyceridemia, metabolic syndrome, and type 2 diabetes mellitus in premature CAD patients. Novel in the present study is the association between LIPA polymorphisms and different metabolic traits. This first time report on the associations of *LIPA* polymorphisms with metabolic traits needs to be confirmed in other studies.

*LIPA* polymorphisms, rs1412444 and rs2246833, were found to be significantly associated with increased *LIPA* expression levels [11]. This increase might enhance intracellular release of fatty acids and cholesterol via the lysosomal route, explaining the role of LIPA in atherosclerosis [19]. On the other hand, the increased LIPA expression may produce an increased LAL-activity, generating an increase in the cholesteryl ester hydrolysis. It is well known that cholesteryl ester hydrolysis participates in the enzymatic modification of LDL particles, transforming them into proatherogenic particles [20]. Only two studies reported previously the effect of the rs1412444 and rs2246833 polymorphisms in *LIPA* expression levels. In these studies, both polymorphisms (rs1412444 and rs2246833) were associated with increased expression levels of LIPA [11,18]. However, gene expression studies performed in monocytes and other cell types might yield different results. Considering that no other studies have reported this functional effect, we decided to perform a functional prediction analysis. Based on SNP functional prediction software (http://snpinfo.niehs.nih.gov/snpfunc.htm), only the rs1412444 polymorphism seems to be functional. In this case, the presence of the *T* allele produces a loss (or reduction) of DNA binding of transcription factors with ETS domain with important consequences on the expression of the lysosomal acid lipase. Conserved ETS DNA-binding domains mediate transcriptional regulation at ETS sequence-containing promoters and contain many phosphorylation sites targeted by various MAP kinases, following exposure to cell stressors or mitogenic stimuli [21]. However, this predicted functional consequences of the rs1412444 *T* allele needs experimental testing. Considering the possible functional effect of these polymorphisms, the association of rs2246833 with premature CAD could be due to linkage disequilibrium with rs1412444.

## Conclusion

In summary, our results replicate two polymorphisms, which were proven to be associated with CAD in Caucasian and Asian populations in Mexican patients with premature CAD. This is the first study that reports the association of *LIPA* polymorphisms with CAD in a non-Caucasian- and non-Asian- origin population, suggesting that rs1412444 and rs2246833 of the *LIPA* gene are shared susceptibility polymorphisms for CAD among different ethnicities. Novel associations of LIPA polymorphisms with metabolic traits were also reported.
