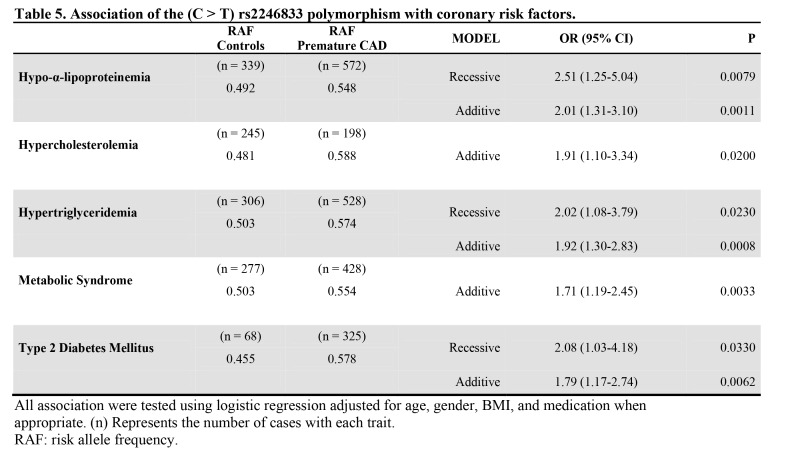# Correction: Single Nucleotide Polymorphisms within *LIPA* (Lysosomal Acid Lipase A) Gene Are Associated with Susceptibility to Premature Coronary Artery Disease. A Replication in the Genetic of Atherosclerotic Disease (GEA) Mexican Study

**DOI:** 10.1371/annotation/f99d1cf3-ad36-4185-bde4-a6bf654ccdf5

**Published:** 2013-12-30

**Authors:** Gilberto Vargas-Alarcón, Carlos Posadas-Romero, Teresa Villarreal-Molina, Edith Alvarez-León, Javier Angeles, Maite Vallejo, Rosalinda Posadas-Sánchez, Guillermo Cardoso, Aida Medina-Urrutia, Eric Kimura-Hayama

There were errors in the typesetting of Tables in the article. Correct versions of the Tables are available below.

Table 1: 

**Figure:**
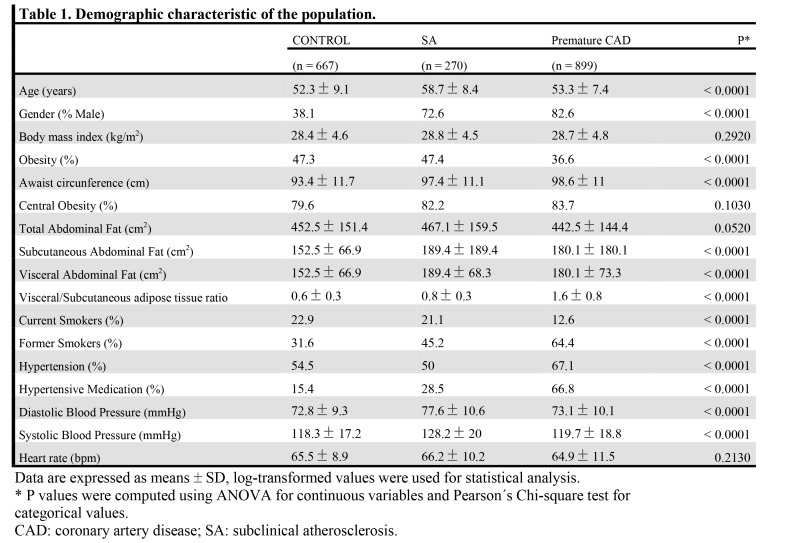


Table 2: 

**Figure:**
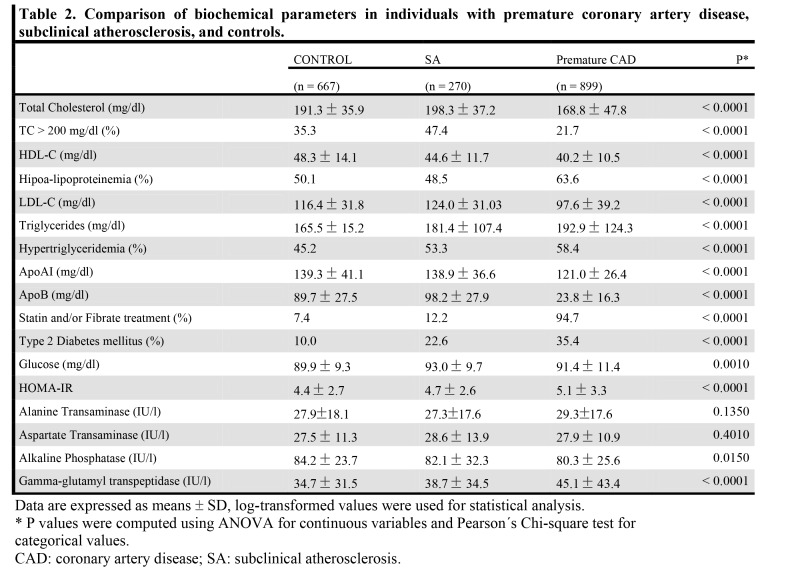


Table 3: 

**Figure:**
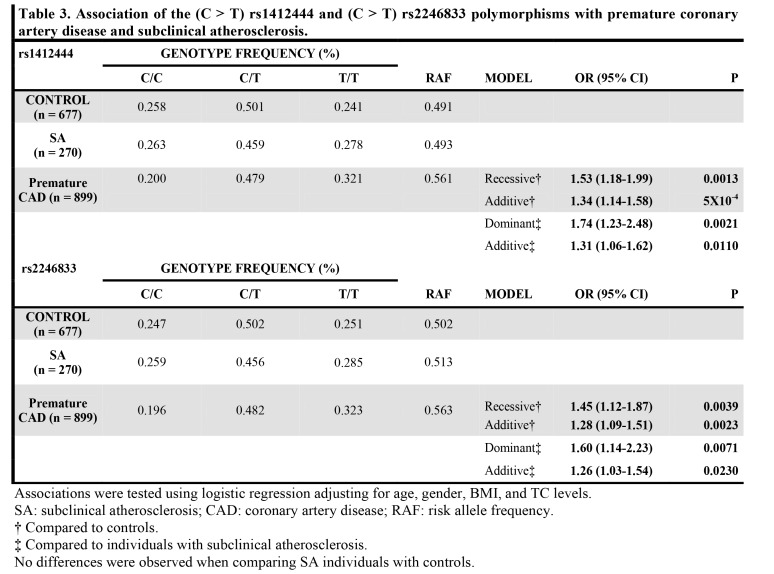


Table 4: 

**Figure:**
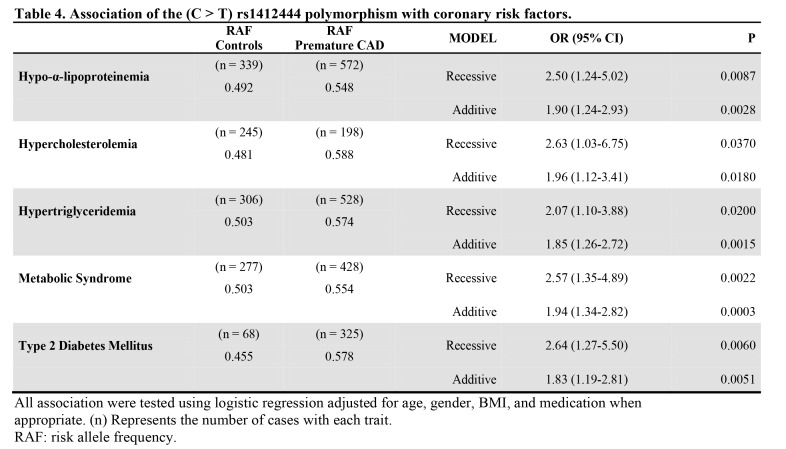


Table 5: 

**Figure:**